# Special Report on the International Medical and Sanitary Exhibition, South Kensington

**Published:** 1881-09

**Authors:** 


### Special Report on the International Medical and Sanitary Exhibition, South Kensington.

"Messrs. Schieffelin & Co., New York.—The articles intended for exhibition by this well-known firm were, unfortunately, lost in the wreck of the steamship "Britannic," so that any complete account of them is impossible as yet. A duplicate series, however, is, we are informed, on the way from America, and it is hoped that by the end of the week the case will be completely filled. The specialty on which Messrs. Schieffelin base their claim to attention is *soluble pills and granules*, samples of which were shown on the opening day of the exhibition. These are most beautifully made pills, coated with an inert, soluble compound, which may be found by trial, to dissolve almost immediately on the tongue, thereby bringing their coated pills on a level, as to efficacy, with the best uncoated articles. In addition, the envelope is perfectly transparent, whence the exact color and appearance of the pill-masses are at once disclosed to the eye, and it becomes an easy matter to distinguish the different pills from one another, and thus prevent mistakes arising, such as may occur when the outside of all the pills employed is the same. By careful manipulation the exact equality of the pills of a kind is maintained, and the materials employed in their manufacture are the best obtainable. Remembering the many pills claiming the approbation of the profession, and the bases on which the claims made depend, it is almost impossible to avoid the conclusion that those of Messrs. Schieffelin combine all the qualities sought in a good pill, while in appearance they yield to none for elegance and shape. They are, moreover, permanently protected, and retain their qualities for years. They are made in four hundred varieties. We hope to speak further, bye-and-by, of the other exhibits prepared by Schieffelin & Co."—*From the London Medical Press and Circular*, July 20, 1881.

